# Stress and health related quality of life of Nepalese students studying in South Korea: A cross sectional study

**DOI:** 10.1186/1477-7525-10-26

**Published:** 2012-03-13

**Authors:** Pratibha Bhandari

**Affiliations:** 1 Division of Nursing Science, College of Health Science, Ewha Womans University, Helen Building, 11-1 Daehyun Dong, Seodaemun gu, Seoul 120-750, South Korea

**Keywords:** Health related quality of life, Stress, Acculturation, Students, Nepal

## Abstract

**Background:**

In recent years there has been a growing trend among students to travel for educational purposes to other countries where there is the possibility of experiencing considerable amounts of stress affecting their physical and mental functioning. The aims of the current study were to investigate the health related quality of life (HRQOL) of Nepalese students studying in South Korea to explore the relationship between HRQOL and perceived and acculturative stress, and to identify the determinants of HRQOL.

**Methods:**

One hundred and thirty students were enrolled in this study. HRQOL was assessed using the Medical Outcomes Study Short Forms (SF-12) questionnaire. Perceived stress and acculturative stress was measured using the Perceived Stress Scale and Acculturative Stress Scale for international students, respectively. Pearson's correlation test and multiple regression analysis were performed.

**Results:**

Perceived stress and acculturative stress were negatively correlated with HRQOL. The highest value in the HRQOL was reported for the vitality subscale and the lowest value was reported for the role-emotional. In the regression model, perceived stress, acculturative stress, relationship with advisor, and marital status accounted for a significant (p < .001) portion of the variance (49%) in the mental component summary of the HRQOL.

**Conclusions:**

The findings of this study indicate that Nepalese students studying in South Korea experience a considerable amount of perceived and acculturative stress, which is negatively related with their HRQOL. Provision of culture specific counseling and orientation programs may benefit the students. The determinants of HRQOL identified in this study were perceived stress, acculturative stress, relationship with advisor, and marital status.

## Background

HRQOL is a multidimensional subjective concept and also an important health determinant [1]. Better HRQOL is vital for normal and productive functioning of an individual. Over the years, several studies have demonstrated the importance of HRQOL assessment among different groups of people, including university students. University life, where major life transition occurs, has often been recognized as a stressful period in one's life that can result in lowered levels of HRQOL. Academic pressures, peer pressure, pressures in relationships, being away from home, and financial concerns during the transition from school to university life have been identified as common stressors among university students [2]. Further in recent years following globalization, there has been an increased trend among the student population to travel to other countries for educational purpose causing a sharp rise in the number of international students in many Western and Asian countries. In the case of international university students, experiences of acculturation and adaptation, racial discrimination, language barriers, home sickness, differences in the educational systems, cultural differences in male-female relationships, and financial difficulties further cause substantial amounts of stress [3-6]. While a certain level of stress can be attributed as a motivating and facilitating factor for academics work and the acculturation process among international students; nevertheless, in most cases, stressors disturb the stability or wellness of the client system causing negative physical and mental health related outcomes. Hence, over the past decades, many researchers and policymakers have carried out research to describe stress and HRQOL among university students [2,7-9]. Previous research on stress among university students has noted the magnitude of stress to vary according to academic year, gender, cultural background, field of study, age, availability of social support, and the student's relationship with their advisor. A cross-sectional study among European medical students by Dahlin et al. revealed that the highest degree of stress was encountered in first year due to academics. Differences among male and female participants was also seen in this study, with female students reporting higher levels of stress [8]. Irrespective of the source of stress, undue stress can foster negative lifestyle practices like drinking alcohol, depression, anxiety, and also suicidal ideations [10,11]. Perceived stress has been identified as an important risk factor for low scores on the mental component of health, indicating frequent psychological distress, and emotional problems causing social and role disabilities. This further deteriorates the HRQOL of university students [12-14]. The prevalence of psychological morbidity among university students is reported to be 21.8% [15].

From the literature review, it can be pointed out that stress during university life leads to poor HRQOL among international/university students, which manifests as poor academic performance, broken social relationships, and disorders like depression. Understanding the HRQOL of international students will enable the host countries to better address the needs of international students thereby improving their academic performance and general satisfaction. In addition, assessment of HRQOL, which is an important determinant of health, will enable the health care providers to reduce healthcare disparity among the growing international student population.

International students account for a large percentage of the reported growth in immigrant population of South Korea. The South Korean immigration service has reported this percentage to be 16.6% in the year 2010. Further, among the student population the largest number reported is of Asian students, among which the number of Nepalese students is 432, including students in regular and specialized courses (e.g., religious studies) [16]. However, studies investigating the HRQOL of any international student group is completely lacking in South Korea. Hence the purpose of the current study was to estimate the perceived stress and acculturative stress of Nepalese students studying in South Korea and to explore the association of HRQOL with stress and other demographic variables. In addition, determinants of HRQOL were also identified.

## Method

### Design

Cross-sectional self administered questionnaire survey

### Participants

**Figure 1:**
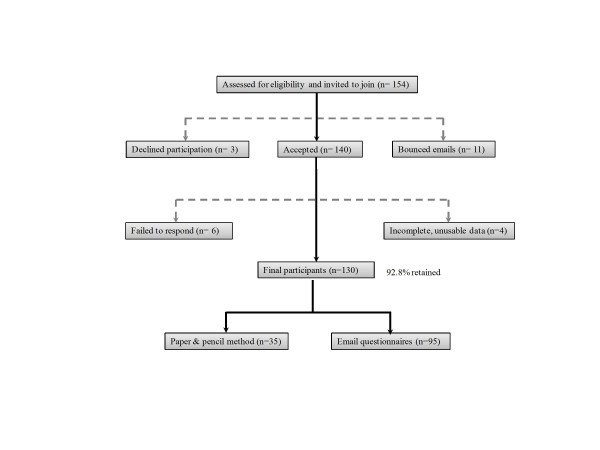
**Study sample recruitment diagram**.

Convenient sampling was used to recruit participants for this study. The baseline information about the students was obtained from the database of Society of Nepalese Students in Korea (SONSIK). Preliminary emails explaining the purpose of the study were sent to all members of SONSIK. The inclusion criteria were: a) Nepalese undergraduate, graduate, or a student pursuing language course in South Korea and b) students who have been residing in Korea for over three months. Participation was voluntary. Ethical approval was obtained from the Institution Review Board (IRB), and informed consent was taken from all participants. Required sample size for hierarchical regression calculated using online statistics calculator was 94 (p = 0.05; desired statistical power = 0.80; and anticipated effect size = 0.15). The number of participants in the current study was 130. The sample recruitment is detailed in Figure 1.

#### Measures

##### General demographic characteristics

General demographic information was obtained using a questionnaire designed by the researcher.

##### Perceived stress

Perceived stress scale (PSS) was used in this study to assess the perceived stress. PSS consists of 14 items marked on a Likert scale of 0 to 4 for each item, in the range of never, almost never, sometimes, fairly often, and very often, respectively. The scores are obtained by reversing the scores on seven positively stated items and summing the scores across all 14 items. The total score ranges was from 0 to 56, with higher scores indicating higher levels of perceived stress [17].

##### Acculturative stress

Acculturative stress was measured using a 36 item acculturative stress scale for international students (ASSIS). The responses are assessed in a Likert scale ranging from 1 (strongly disagree) to 5 (strongly agree). The total score for the ASSIS ranges from 36 to 180, with higher scores indicating greater acculturative stress [18].

##### Health related quality of life

The SF-12 version 2 questionnaire [19] was used to assess the HRQOL. The SF-12 includes twelve items that represent eight health concepts, namely physical functioning, role limitations (physical), bodily pain, general health, vitality, social functioning, role limitation (emotional), and mental health. The total score for the SF-12 is computed as a mental health component summary (MCS) and a physical health component summary (PCS). Higher scores on PCS and MCS indicate having a better self-reported health-related quality of life.

#### Data collection

The period for data collection was from March 7, 2011 to May 6, 2011. An email explaining the purpose of the study was sent initially to 154 Nepalese students. Students willing to voluntarily participate in the study were followed up further with the questionnaires. For students residing in Seoul and nearby provinces, paper and pencil method was used for data collection, whereas for students studying/residing outside of Seoul, the questionnaires were sent as an email attachment and follow up was conducted by phone call (Figure 1). The filled questionnaires were sent back to the researcher by email within a week.

#### Data analysis

Data were analyzed using the Predictive Analytic Software (PASW Statistics) 18 program and Quality Metric Health Outcomes™ Scoring Software 4.0 [19]. The general characteristics of the participants and the stress scores were analyzed using descriptive statistics. Perceived stress, acculturative stress, and the HRQOL of the participants were compared on general demographic variables using a *t*-test and ANOVA. The Pearson's correlation test was used to explore the association of HRQOL with stress and other demographic variables. The MCS and PCS score for the HRQOL was computed using Quality Metric Health Outcomes™ Scoring Software 4.0. Multiple regression analysis was then conducted to identify determinants of the HRQOL of Nepalese students studying in South Korean universities. The level of significance was set at *p = < 0.05*, 95% CI.

## Results

The general demographic characteristics of the participants are presented in Table 1.

**Table 1:** General demographic characteristics (n = 130)

Variables	n	%
Age		
20-29	65	50.0
30-39	53	40.8
40-50	12	9.2
Gender		
Male	103	79.2
Female	27	20.8
Religion		
Hindu	113	86.9
Christian	2	1.5
Buddhists	2	1.5
Others	1	.8
None	12	9.2
Marital Status		
Married	73	56.2
Single	57	43.8
Living with spouse (n = 73)		
No	26	35.6
Yes	47	64.3
Level of Study		
Undergraduates	7	5.4
Masters	32	24.6
PhD	91	70.0
Major		
Engineering	28	21.5
Natural Science	84	64.6
Humanities & Social Science	13	10.0
Interdepartmental Programs	5	3.8
Financial Support		
Self	6	4.6
Korean Government scholarship	29	22.3
Nepal Government scholarship	1	.8
University scholarship	43	33.1
Research assistantship	24	18.5
Both (University & Research assistantship/Government & Research assistantship)	26	20.0
Others	1	.8
Years spent in Korea		
Less than 1 year	13	10.0
1-3 years	68	52.3
3-5 years	34	26.2
More than 5 years	14	10.8

Perceived stress, acculturative stress, and HRQOL were measured using valid and relaible instruments. The Cronbach's alpha for PSS, ASSIS, and SF-12 were .79, .90, and .80, respectively. The mean perceived stress score and acculturative stress scores of the participants were 21.57 ± 7.43 and 87.02 ± 18.97, respectively. The difference in the PSS and ASSIS scores between male and female participants was not statistically significant. In the current study, the mean PCS and MCS score of the participant was 51.42 ± 7.25 and 49.40 ± 8.93, respectively, with the female subset reporting lower scores in both PCS and MCS. Forty five percent of the sample scored below the general US population norm in the PCS while 40% of the sample scored below the general US population norm in the MCS. The highest value was reported for the vitality subscale, followed by the physical functioning; and the lowest value was reported for the role-emotional.

Comparison of perceived stress, acculturative stress, and PCS and MCS of the HRQOL was made on certain demographic characteristics, namely gender, marital status, living status in Korea, place of residence, age group, level of study, and major. Significant difference in MCS was observed between married and single Nepalese students (*p *= .05). The results are displayed in Table 2.

**Table 2:** Comparison of perceived stress, acculturative stress, PCS and MCS based on general demographic characteristic

			Perceived Stress			Acculturative Stress			Physical Component Summary (PCS)			Mental Component Summary (MCS)		
			
Characteristics	Category	N (%)	Mean ± SD	t or F	*p*	Mean ± SD	t or F	*p*	Mean + SD	t or F	*p*	Mean ± SD	t or F	*p*
Age Group	20-29	65(50)	22 ± 8.6	.25	.77	87.2 ± 19.6	.51	.59	50.7 ± 7.76	.63	.52	48 ± 9.21	1.46	.23
	30-39	53(41)	21.1 ± 6.2			88.4 ± 19.8			51.9 ± 6.78			50.5 ± 8.94		
	40-50	12(9)	20.8 ± 3.7			82 ± 19.9			52.6 ± 6.58			51.4 ± 6.46		
Gender	Male	103(79)	20.9 ± 7.25	-1.90	.06	86.7 ± 19.5	-.51	.60	51.9 ± 6.94	1.6	.09	49.7 ± 8.94	.84	.39
	Female	27(21)	23.9 ± 7.73			89 ± 20.39			49.3 ± 8.16			48.1 ± 8.94		
Marital Status	Married	73(56)	21.1 ± 6.8	-.81	.42	88.8 ± 19.0	1.03	.30	50.4 ± 7.67	-1.71	.09	50.7 ± 8.49	1.95	.05*
	Single	57(44)	22.1 ± 8.2			85.2 ± 20.3			52.6 ± 6.54			47.6 ± 9.25		
Living status in	With spouse	47(64)	20.8 ± 7.3	-.45	.65	89.4 ± 19.4	.34	.73	50 ± 8.27	-.65	.51	50.6 ± 8.39	-.07	.93
Korea	Without spouse	26(20)	21.6 ± 5.8			87.8 ± 18.6			51.2 ± 6.54			50.8 ± 8.86		
Level of study	Undergraduate	7(5)	53 ± 2.7	.39	.67	83.7 ± 14.3	1.59	.20	53 ± 2.70	.90	.40	48 ± 7.79	.10	.90
	Masters	32(25)	52.5 ± 7.4			82.2 ± 18.1			52.6 ± 7.42			49.1 ± 7.96		
	PhD	91(70)	50.8 ± 7.4			89.2 ± 20.3			50.8 ± 7.41			49.5 ± 9.39		
Major	Engineering	28(21)	21.3 ± 8	.21	.88	89.4 ± 15.1	.20	.89	53.1 ± 6.30	.97	.40	47.9 ± 10.0	.33	.80
	Natural Science	84(65)	21.8 ± 7.6			86.9 ± 21.2			50.6 ± 7.55			49.8 ± 8.82		
	Humanities& Social	13(10)	20.6 ± 5.3			84.3 ± 20.2			52 ± 7.72			49.9 ± 8.24		
	Science	5(4)	19.5 ± 3.3			87 ± 16.9			53.1 ± 5.43			48.9 ± 7.14		
	Interdepartmental programs													
Place of	Seoul	35(27)	21.6 ± 7.8	.06	.94	86 ± 17.1	-.44	.66	53.2 ± 7.71	1.75	.08	48.9 ± 7.65	-.32	.74
residence	Out of Seoul	95(73)	21.5 ± 7.3			87.7 ± 20.5			50.7 ± 7.0			49.5 ± 9.39		

*p = .05

Perceived stress was found to correlate negatively with the physical (r = -0.189 *p *= 0.05) and the mental (r = -0.642; *p *= 0.01) component summary of the HRQOL. Acculturative stress was also negatively correlated with the HRQOL (PCS: r = -0.362; *p *= 0.01; MCS: r = -0.442; *p *= 0.01). Both perceived stress and acculturative stress were highly correlated with the mental component summary of the HRQOL. The correlation matrix is shown in Table 3.

**Table 3:** Relationship between HRQOL and study variables

		1	2	3	4	5	6	7
1	PCS							
2	MCS	-.051						
3	Gender	-.147	-.075					
4	Relationship with advisor	.135	.390**	-.084				
5	Marital Status	.149	-.17	-.070	-.005			
6	Perceived Stress	-.189*	-.642**	.169	-.315**	.073		
7	Acculturative Stress	-.362**	-.442**	.054	-.333**	-.109	.463**	

* p < .05 level (2-tailed)

** p < .01 level (2-tailed)

Hierarchical multiple regression analysis was conducted to identify determinants of HRQOL. The analysis was conducted separately for the MCS and PCS as outcome variable. The predictor variables used for the model were perceived stress, acculturative stress, gender, relationship with advisor, and marital status, which were entered in three blocks (models). The demographic variables (*gender and marital status*) were entered in the first block; *relationship with advisor *was entered in the second block followed by *perceived stress and acculturative stress *in the third block. With the MCS as outcome variable, the first model accounted for 4% of the variance. The addition of the variable *relationship with advisor *in the second model caused an increase of 14% in the variance (*p *= < .001). *Perceived stress and acculturative stress *were added in the third block, which accounted for an additional 31% of the variance in the MCS (*p *= < .001). The final model accounted for 49% of the variance in the MCS. In the final model, *perceived stress, acculturative stress, relationship with advisor*, and *marital status *accounted for a significant portion of the variance in the MCS. With the PCS as an outcome variable, the final model accounted for 15% of the variance in the PCS (*p *= < .05). However, only *acculturative stress *accounted for a significant portion of the variance in the final model. The result of the hierarchical regression analysis is displayed in Table 4 and 5.

**Table 4:** Summary of hierarchical regression analysis for variables predicting MCS of HRQOL

	Model 1			Model 2			Model 3		
	
Variables	B	SE B	β	B	SE B	β	B	SE B	B
Gender	1.91	1.97	0.09	1.20	1.82	.05	-.46	1.47	-.02
Marital Status	3.15	1.60	.18*	3.08	1.49	.17*	2.68	1.20	.15*
Relationship with advisor				3.99	.86	.39**	1.80	.73	.17*
Perceived Stress							-.60	.10	-.50**
Acculturative Stress							-.08	.03	-.19*
R^2^	.04			.18			.49		
F for change in R^2 ^(df)	2.28(2,121)			8.10(3,120)**			22.95(5,118)**		

*p <.05, **p <.001

**Table 5:** Summary of hierarchical regression analysis for variables predicting PCS of HRQOL

	Model 1			Model 2			Model 3		
	
Variables	B	SE B	β	B	SE B	β	B	SE B	β
Gender	2.43	1.60	.13	2.24	1.60	.12	2.11	1.54	.12
Marital Status	-2.03	1.30	-.14	-2.05	1.30	-.14	-1.67	1.25	-.11
Relationship with advisor				1.05	.75	.12	.10	.77	.01
Perceived Stress							-.02	.10	-.02
Acculturative Stress							-.11	.03	-.31*
R^2^	.04			.06			.15		
F for change in R^2 ^(df)	2.58 (2,121)			2.39 (3,120)			4.13 (5,118)*		

*p <.05

## Discussion

In the current study, the majority of the participants were male and married. Among the married participants, 64.3% were living together with their spouse in South Korea. This is in contrast to some study findings where most of the international student sojourners were single [20,21]. Each cultural group is unique, with specific needs and characteristics [22]; the higher percentage of married Nepalese students in this study can be attributed to Nepalese culture, where youths get married at an early age, and married couples are encouraged to live together [23,24]. Additionally, the majority of participants in this study were enrolled in Department of Natural Science and were pursuing doctoral degrees in South Korea. Unlike most English speaking countries, where many students pursue humanities, social science, and health related studies along with technical courses [20,25], this study showed that most of the Nepalese students were enrolled in technical fields like engineering and natural science. This can be due to the language barrier, which plays a central role in academics and the acculturative process [26]. Analysis of the demographic data also revealed that only 4.6% of the Nepalese students were supporting their educational expenses themselves (parents) as most of the students were receiving educational support either from the government or their respective universities/professors. This is in contrast to the finding reported by Khawaja and Dempsey (2008) where 67.1% of the international students received financial support through their parents. Since Nepal is one of the poorest country in the world, with 55% of the population living below the international poverty line of US $ 1.25 per day [27], the majority of students are highly dependent on the scholarships provided by the host government and or the host universities.

In the current study, the mean perceived stress score of the participants was lower compared to the doctor of pharmacy students (26.5 ± 8), as reported by Marshall et al. (2008). PSS measures subjective evaluation of the stressfulness of a situation, which can be influenced by daily happenings and major life events [17]. The comparatively low mean perceived stress reported in the current study can be due to the fact that the new semester begins in March in South Korea, and the majority of the data were collected between March through mid April when the academic load is apparently low. The mean perceived stress scores for the female subset in the current study was higher compared to those of the male subset, though the difference between male and female was not statistically significant. This finding is in agreement with reported literature from the USA, [10] Pakistan [28], and Egypt [29]. However, it should be pointed out that even though this finding is in line with the previously reported literature, it could also be because of the cultural influence where Nepali men, who are generally the dominant figure in the society, are hesitant to objectively report their perceived stress. This finding can be further validated through qualitative studies. No significant difference was observed in acculturative stress scores between males and females. A study conducted by Constantine et al. with a diverse group of international students has reported acculturative stress among Asian students to be significantly lower compared to Latin American students [6]. The high prevalence of acculturative stress among Nepalese students in this study could partly be due to the fact that Korea is not an English speaking country.

The PCS and MCS scores were lower in the female subset, which is similar to findings reported from previous literature [10,30-32]. For the total sample, the highest value was reported for the vitality subscale, followed by physical functioning; and the lowest value was reported for the role-emotional. This is in contrast to findings reported by Arslan et al., where the lowest score was reported for the vitality subscale [15]. The finding reported with Belgrade university students [30] is similar; it also mentions the lowest score on the vitality subscales. A study conducted among a Nepalese sample in Kathmandu by Sakai et al. also reports high scores for physical functioning, body pain, and vitality among the Nepalese male population [32]. Even though there is a general assumption about Nepalese (Gurkhas) being physically strong and brave, there is no scientific finding to support or refute this. Further research can be done to validate higher scores reported for physical functioning and vitality. Sakai, et al. also reports higher scores on the physical functioning subscale in "higher caste groups" when compared to "lower caste groups"[32]. Difference in the HRQOL based on caste/ethnic group, being a culturally sensitive issue among the Nepalese, was not explored in this study.

Both perceived stress and acculturative stress was negatively correlated with the physical and mental component summary of the HRQOL, which is in agreement with previously reported literature [2,10,33].

In order to identify the determinants of the HRQOL, the analysis was conducted separately for the MCS and PCS as outcome variable. The variables used for the regression model were perceived stress [10,15,30], acculturative stress [3,14,25,34], gender [29,31,33], relationship with advisor [14], and marital status [14,35]. Perceived stress emerged as the strongest determinant of the mental component of the HRQOL. This is in line with the findings reported by Bovier et al. [12]. In the case of PCS, only acculturative stress accounted for a significant portion of the variance in the HRQOL. In the current study, lack of significant contribution by sex in the final regression model could be due to the small sample size of the female participants (n = 27).

As mentioned previously, this study is the first assessing stress and HRQOL in a group of international students in South Korea. Hence, it can be looked upon as an important milestone for planning future research. However, this study was subject to some limitations. First, although the instruments used in this study were proven to have good reliability and validity with other populations, participants in this study might have had difficulty in understanding the English questionnaires. Additionally, the length of the questionnaires might have overwhelmed the participants affecting the internal validity. Hence, the results should be interpreted with caution. International students seeking enrollment in South Korean universities are expected to have either English or Korean language proficiency validated by high scores in language proficiency standard tests like Test of Proficiency in Korean Language (TOPIK) or TOEFL, IELTS, TOEIC, etc., and, hence, it was assumed that the students would have no difficulty using self-reported English questionnaires. Also, the Department of Immigration in South Korea reports the total number of Nepalese students studying in South Korea as 432, but only 140 Nepalese students were accessible for this study. This may have introduced selection bias, and, hence, the observed result may not be representative of all Nepalese students in general. Second, higher perceived stress and acculturative stress among international students are related with negative affects, such as sadness, depression, and suicidal ideations [11,12,36,37]. In this study, variables such as depression, anxiety, negative lifestyle practices, and social support, which are commonly reported to be associated with stress levels and as affecting the HRQOL among university students, were not assessed. Additionally, negative and positive coping strategies, such as use of religion [38], drinking alcohol [10], planning, positive reframing of one's thoughts, and denial [9] have also been reported in the literature. In this study, stress coping strategies adopted by the students were not assessed. In order to elucidate the effect on HRQOL, further studies must be planned with the inclusion of both stress and coping mechanism adopted by the students. Finally, since perceived stress and acculturative stress vary with time period a onetime measurement as a cross-sectional survey can be looked as a limitation.

## Conclusion

The results from this study support the assumption that Nepalese students studying in South Korea experience a considerable amount of perceived and acculturative stress, which significantly affects their HRQOL. The findings indicate that perceived stress, acculturative stress, relationship with advisor, and being single nonmarried educational sojourners are determinants of the HRQOL of Nepalese students. Based on these findings it is recommended that measurement of perceived stress among international students should be carried out periodically at all universities. In addition, culture specific counseling and orientation programs must also be planned at each university.
